# Culture-Independent Identification of *Mycobacterium avium* Subspecies *paratuberculosis* in Ovine Tissues: Comparison with Bacterial Culture and Histopathological Lesions

**DOI:** 10.3389/fvets.2017.00232

**Published:** 2017-12-22

**Authors:** Kamal R. Acharya, Navneet K. Dhand, Richard J. Whittington, Karren M. Plain

**Affiliations:** ^1^Faculty of Science, Sydney School of Veterinary Science, The University of Sydney, Camden, NSW, Australia; ^2^School of Life and Environmental Sciences, University of Sydney, Sydney, NSW, Australia

**Keywords:** *Mycobacterium avium* subspecies *paratuberculosis*, abattoir surveillance, quantitative PCR, strain typing, sheep, tissue

## Abstract

Johne’s disease is a chronic debilitating enteropathy of ruminants caused by *Mycobacterium avium* subspecies *paratuberculosis* (MAP). Current abattoir surveillance programs detect disease *via* examination of gross lesions and confirmation by histopathological and/or tissue culture, which is time-consuming and has relatively low sensitivity. This study aimed to investigate whether a high-throughput quantitative PCR (qPCR) test is a viable alternative for tissue testing. Intestine and mesenteric lymph nodes were sourced from sheep experimentally infected with MAP and the DNA extracted using a protocol developed for tissues, comprised enzymatic digestion of the tissue homogenate, chemical and mechanical lysis, and magnetic bead-based DNA purification. The extracted DNA was tested by adapting a previously validated qPCR for fecal samples, and the results were compared with culture and histopathology results of the corresponding tissues. The MAP tissue qPCR confirmed infection in the majority of sheep with gross lesions on postmortem (37/38). Likewise, almost all tissue culture (61/64) or histopathology (52/58) positives were detected with good to moderate agreement (Cohen’s kappa statistic) and no significant difference to the reference tests (McNemar’s Chi-square test). Higher MAP DNA quantities corresponded to animals with more severe histopathology (odds ratio: 1.82; 95% confidence interval: 1.60, 2.07). Culture-independent strain typing on tissue DNA was successfully performed. This MAP tissue qPCR method had a sensitivity equivalent to the reference tests and is thus a viable replacement for gross- and histopathological examination of tissue samples in abattoirs. In addition, the test could be validated for testing tissue samples intended for human consumption.

## Introduction

Ovine Johne’s disease (OJD) is a chronic debilitating gastroenteritis of sheep caused by *Mycobacterium avium* subspecies *paratuberculosis* (MAP). OJD-specific enteric lesions cause protein loss and chronic malabsorption of protein (1, 2) leading to a protracted wasting condition in the affected sheep. Consequently, OJD-infected sheep can have poor welfare status and reduced production traits, including quality of the carcass and fleece (3). Control of OJD has been attempted by means of flock vaccination, which is effective to an extent (4–6); however, this strategy has failed to achieve a complete cessation of fecal shedding (5, 6). Fecal shedding of the bacteria by the vaccinated sheep could become a continued source of infection in the flock through faeco-oral transmission to other susceptible animals. Therefore, implementation of a suitable OJD surveillance program is essential for detection and control of MAP spread, both in vaccinated and unvaccinated flocks.

Ovine Johne’s disease surveillance activities include collection and testing of a range of samples from suspected sheep. In Australia, to confirm infection in suspected animals and flocks, fecal culture and/or fecal quantitative PCR (qPCR) (7) may be performed, whereas postmortem confirmation is attained by tissue culture and examination of histopathological lesions within tissue samples such as intestine and mesenteric lymph nodes (MLNs) collected at abattoirs participating in the National Sheep Health Monitoring Program or on farm (8–10).

For confirmation of MAP infection, culture of fecal samples is less reliable than tissue culture of the small intestines and MLN, the major predilection sites of MAP, in both clinically and subclinically affected animals (11–15). This is due to the intermittent fecal shedding of MAP (16). Furthermore, passive fecal shedding of MAP has been noted in animals irrespective of their infection status and animals exposed to MAP showing positive fecal sample test results may never become infected or may clear the infection (17, 18). Like fecal samples, biopsies could also be collected from live animals for the confirmation of MAP infection in sheep (17, 19, 20). Testing of tissue samples for confirmation of MAP infection could also have an application for human health, as MAP has been suggested to be the causative agent of Crohn’s disease in humans (21, 22). MAP has been detected in biopsies from the intestinal tissues of humans (23), including pediatric Crohn’s disease patients (24). Human exposure could be associated with the consumption of animal tissues from infected animals that can contain MAP, due to dissemination of bacteria during systematic infection or through fecal contamination during slaughter procedures (25–28). Hence, a suitable tissue sample collection and testing approach could significantly contribute to the detection of MAP infected sheep.

The culture of tissue homogenate is considered the gold standard test for confirmation of MAP infection in animals. This test has been reported to be more sensitive than examining histological lesions, Ziehl–Neelsen staining and/or immunohistochemistry (29), and fecal culture (14). However, the use of decontaminants reduces the sensitivity of this method (30) and the long incubation time is a further limitation (31). Therefore, development and validation of alternative techniques is essential, especially if the test result is intended for surveillance purposes. Culture-independent methods such as qPCR that require mycobacterial DNA isolated directly from the sample have been used successfully to diagnose nontuberculous mycobacterial infection (32) and to perform whole genome sequencing of *Mycobacterium tuberculosis* (33). Similar approaches could be used to diagnose as well as rapidly identify the strain and type of MAP, thereby aiding in molecular epidemiology to identify the source of infection. Diagnostic tests based on qPCR are preferred, owing to the speed and high-throughput nature of these assays. In addition, there is some evidence that qPCR may perform better than tissue culture to detect MAP in diaphragm muscle samples of cattle (25).

Several conventional PCR or qPCR methods have been reported for the detection of MAP DNA directly from intestine, MLN, meat, and formalin-fixed paraffin-embedded intestinal tissues (34–41). There are few studies that focus on testing sheep and goat tissues (38, 41, 42). Although some studies have compared qPCR with culture methods using bovine and caprine tissue samples (40, 42), a comparative study using ovine tissue samples is lacking.

Therefore, the objectives of this study were to (a) test the tissue homogenates (intestinal and MLN) from MAP-exposed sheep using a qPCR method with suitable sensitivity, (b) compare qPCR with other relevant diagnostic techniques such as tissue culture and examination of histopathological lesions of the tissue sections, and (c) assess the potential for direct strain typing by means of a restriction endonuclease analysis (REA) IS*1311* method.

## Materials and Methods

### Sample Collection

The tissue samples originated from a trial, in which the animals were infected using validated infection model (43). Approval from the University of Sydney Animal Ethics committee was obtained before the commencement of this study. In brief, approximately 5-month-old lambs received a total dose of 2.74 × 10^9^ viable cells of MAP (S strain, Telford 9.2) orally over a period of 1 month. Necropsy of the animals was performed at 14 months post-inoculation or earlier if there was evidence of significant loss of weight (>10% in a month) or death. Intestinal sections (terminal ileum and middle jejunum) and MLN sections (posterior and middle jejunal MLN) of these sheep were collected at necropsy and stored at −80°C before culture.

For each animal, pooled tissue homogenates of intestinal tissues and MLN tissues were prepared separately. Representative sections of tissues amounting to 2–5 g were finely chopped with a sterile scalpel blade and added to an 80–100 mL stomacher bag (Interscience) containing approximately an equal amount of sterile 0.85% w/v sodium chloride solution. Homogenization was achieved by stomaching at the highest speed in a stomacher (Minimix, Interscience) for 2 min. The homogenate was transferred to 4-mL screw capped tubes avoiding any obvious tissue pieces. Two milliliters of tissue homogenate were used for tissue culture and the rest was stored at −80°C for qPCR. In total, 107 intestinal and 107 MLN homogenates were tested corresponding to 107 MAP-exposed animals. The histopathological test result of intestinal samples of five animals and MLN samples of six animals were not available; these samples were excluded when comparing qPCR with histopathological lesions.

### Bacterial Culture

The culture of MAP in tissue homogenates was performed in M7H9C liquid culture media and the growth was confirmed by qPCR, as previously described (44, 45). Briefly, 2 mL of tissue homogenate was transferred to sterile polycarbonate tubes containing 25 mL of 0.75% (w/v) hexadecylpyridinium chloride (HPC), mixed by inverting the tubes and incubated in a dark cupboard at room temperature for 72 h. Without disturbing the HPC in the tubes, 100 µl of the precipitate was transferred to 6 mL of M7H9C liquid media and incubated at 37°C for 12 weeks. MAP growth was confirmed by testing DNA extracted from 200 µL of the culture medium using IS*900* qPCR.

### Tissue Histopathological Examination

Tissue histopathological examination was performed as reported previously (46). For this study, results from the examination performed previously were accessed. In brief, a 5-µm section of formalin-fixed paraffin-embedded tissue sample was microscopically examined following staining with hematoxylin and eosin, and Ziehl–Neelsen method. The lesions were scored by an experienced pathologist (R. Whittington) following the criteria established by Pérez et al. (47). The intestinal lesions were graded from 0 to 3. The grade 3 lesion was further classified into paucibacillary (3a), multibacillary (3b), or severe paucibacillary (3c) depending on the lesion and number of acid-fast bacteria present within the lesion. The lesions in MLNs were graded as mild focal (1), mild multifocal (2), or severe multifocal to diffuse (3). If an animal showed a range of lesions, the highest grade of the lesion score was considered for the analysis. An animal was considered to be positive if the lesion score was ≥1.

### Detection of MAP DNA in Tissue Homogenates

#### DNA Extraction

The tissue homogenates were thawed and vortexed. A 1-mL aliquot of the homogenate was transferred to 1.5-mL sterile screw cap tubes (Interpath services). To 1,000 µL of the homogenate, 20 µL of proteinase K (40 mg/mL solution, Astral) was added. The enzymatic action was facilitated by incubating in a heat block (Ratek) at 50°C for 1 h. Vortexing for 20 s was performed following the addition of proteinase K at the end of the incubation time.

Following enzymatic digestion, the suspension was centrifuged at 15,000 × *g* for 15 min and the supernatant was discarded. The pellet was dissolved in lysis buffer (597 µL buffer RLT and 2.8 µL carrier RNA, Qiagen) and transferred to bead tubes containing 0.3 mL of 0.1 mm diameter silica/zirconium beads in 2-mL screw cap tubes. Bead beating was done twice at 30 Hz for 100 s (TissueLyser, Qiagen) with a change in alignment by 180° between two bead beatings, followed by centrifugation at 16,000 × *g* for 3 min. The supernatant was transferred to 1.5 mL LoBind DNA flip-top tubes (Eppendorf) and stored at −20°C.

#### DNA Purification

DNA was purified using the Biosprint^®^ 96 One-for-all Vet kit (Qiagen). Briefly, 400 µL of the supernatant obtained above was transferred to a 96-well lysate plate containing 40 µL proteinase K (Qiagen) to which 300 µL of magnetic bead suspension comprising 25 µL MagAttract suspension G (Qiagen) in 300 µL isopropanol (Sigma-Aldrich) was added. Magnetic bead-based purification of DNA was performed using an automated platform (MagMAX Express-96; Life Technologies), which included transfer of the bead-bound DNA through three wash steps using two different wash buffers (Buffer AW1 and RPE, Qiagen) contained in separate 96-well plates (Qiagen), air drying of any wash buffer residue and DNA elution in Buffer AVE (Qiagen). The eluted DNA was stored at −80°C before use. The DNA extract was tested neat and also as a fivefold dilution in Buffer AVE (Qiagen).

#### IS*900* qPCR

Quantification of MAP genomic DNA in the DNA extracts from the tissues was performed as previously described (7). The final volume of 25 µL of reaction mixture comprised 5 µL template DNA, 250 nM final concentration of each forward [MP10-1 (5′-ATGCGCCACGACTTGCAGCCT-3′)] and reverse [MP11-1 (5′-GGCACGGCTCTTGTTGTAGTCG-3′)] primers (48) and 12.5 µL of SensiMix SYBR Low-ROX qPCR mastermix (Bioline). The amplification was performed (initial denaturation at 95°C for 8.5 min; 40 cycles of denaturation at 95°C for 15 s, annealing at 68°C for 30 s, and extension at 72°C for 1 min, and melt curve analysis from 55 to 95°C) using an Mx3000P real-time PCR instrument (Stratagene, Agilent). The quantification of MAP DNA was performed by constructing a standard curve using 10-fold serially diluted MAP genomic DNA, ranging from 10 to 0.001 pg/reaction, included in each DNA amplification plate. The acceptance criteria for a positive test result were (i) MAP-specific amplification (melt temperature in the range of 89.1 ± 1.5°C) and (ii) MAP DNA quantity of ≥0.0005 pg/reaction, which is the analytical limit of detection of the qPCR (7).

### Strain Typing Using a Culture-Independent Method

Culture-independent strain typing was performed using DNA extracted directly from the tissues. For this purpose, an IS*1311* PCR followed by REA of the PCR product was performed as described (49). Briefly, 50 µL PCR reactions comprised 5 µL of DNA, 250 ng each of forward M56 (GCGTGAGGCTCTGTGGTGAA) and Reverse M119 (ATGACGACCGCTTGGGAGAC) primers, 2 U of *Taq* polymerase (Roche) and 200 µM of each dNTP in reaction buffer. Amplification conditions were one cycle of denaturation at 94°C for 3 min followed by 37 cycles of 94°C for 30 s, 62°C for 15 s, and 72°C for 1 min. The PCR products were evaluated by electrophoresis at 100 V in 2% agarose gels stained with Redsafe dye (INtRON Biotechnology) with molecular weight marker size VIII (Roche). PCR product was stored at −20°C before REA, performed by digestion with two enzymes (*Mse* I and *Hinf* I, New England Biolabs) for 2 h at 37°C according to the manufacturers’ instructions. Visualization of the products (Gel Doc, Bio-Rad) post-enzymatic digestion was performed by electrophoresis at 100 V in 3% agarose gels stained with Redsafe dye.

### Case Definitions

An animal was considered to be infected if the tissue culture result of the pooled intestinal homogenate (terminal ileum, middle jejunum) and/or pooled MLN homogenate (posterior and middle jejunal MLN) was positive.

An animal was considered to be diseased if there was a histopathological lesion score of ≥1 in any of the intestinal and/or lymph node sections examined (47). For this purpose, mid to terminal ileum and proximal jejunum and ileocecal, posterior jejunal and mid proximal jejunal lymph node samples were examined.

A positive qPCR result corresponded to a MAP-specific DNA quantity of ≥0.0005 pg/5 µl of the DNA extracted from 1 mL of tissue homogenate.

### Statistical Analysis

#### Comparison of qPCR with Tissue Culture and Histopathology

The numeric result from qPCR was categorized into positive and negative categories according to the cut-point of ≥0.0005 pg, as previously described (7). The categorical results were used to test the agreement between the qPCR test result with reference tests (tissue culture and histopathological lesions). The difference between detection rates of qPCR and reference tests was evaluated by undertaking the McNemar’s chi-square test. If the difference was not significant, the agreement between qPCR with reference tests was measured by means of Cohen’s kappa coefficient. The relative sensitivity of tissue qPCR was computed as a ratio of the number of positive test results from tissue qPCR to the number of positive results by the reference test.

Furthermore, ordinal logistic regression analysis was performed to assess the relationship between log_10_ DNA quantified by tissue qPCR and histopathological lesion score of the tissues. For this analysis, lesion score 0 and 1 were merged together and treated as lesion score 1 as there were insufficient observations with lesion score 1. In order to perform the log_10_ transformation, a very small value was added to the samples with zero DNA quantity. Type of tissue (intestine and MLN) was included as a factor in the multivariable model.

The discriminatory ability of numeric qPCR results to differentiate the test positive and negative results of tissue culture and/or histopathology was assessed by means of receiver operating characteristic (ROC) curve analysis and calculation of the area under the curve (AUC). The DNA quantity obtained by tissue qPCR was used to conduct the ROC curve analysis.

#### Comparisons between Tissue Types

The mean log_10_ DNA values quantified by qPCR of intestinal and MLN homogenates were assessed using scatter plots and correlation coefficients. The overall difference was also compared using a Bland–Altman plot, which plotted the difference in log_10_ DNA quantity versus average log_10_ DNA quantity. The DNA quantity was log_10_ transformed after adding a very small value (0.0000001 pg) to the quantified DNA.

A two-sided *p*-value <0.05 was considered statistically significant for all analyses reported in this manuscript. The statistical analyses were conducted using GenStat v 16.2.11713 (VSN International Ltd., Hemel Hempstead, UK) and the graphs were prepared using GraphPad Prism 7 for Windows, Version 7.02 (GraphPad Software, Inc., La Jolla, CA, USA).

## Results

The experimentally MAP-exposed sheep included in the study (*n* = 107) comprised a range of sheep breeds: the majority were Border Leicester (33.7%), followed by White Suffolk cross Merino (33.7%), Poll Dorset (16.9%), and Merino (15.9%) breeds of sheep. The animals were not vaccinated, but shared the same paddock as a group of Merino sheep that were vaccinated against paratuberculosis with Gudair^®^. Male and female animals were almost equally represented in the study.

### General Performance of the qPCR Test

The MAP-specific qPCR method detected 59.9% of intestinal homogenates and 65.5% of MLN homogenates from the exposed sheep as positive for the presence of MAP DNA.

A few of the DNA extracts appeared to contain high amounts of initial MAP DNA, as determined by the raw baseline fluorescence before amplification. Fivefold dilutions of the DNA extracts were also subjected to qPCR with specific amplification seen in both neat and diluted samples. The threshold cycle (*C*_T_) values for such samples was determined within the exponential phase of the amplification curve after assessment of the raw amplification plot (data not shown).

### Ability of the Tissue qPCR to Confirm MAP Infection in Animals with Clinical Signs and Gross Lesions of Paratuberculosis

The qPCR method was successful in detecting animals that exhibited clinical signs and gross lesions consistent with OJD. All of the 25 of animals that had clinical signs suggestive of the disease (25/107; 23.4%) were confirmed as positive for MAP DNA by qPCR of intestinal homogenates. Histopathological lesion examination and MAP tissue culture of the intestine and MLN at necropsy also confirmed that these were true clinical cases. However, one of the clinical cases was negative by qPCR of the MLN alone. Similarly, qPCR confirmed 37 of the 38 (97.4%) animals that showed gross lesions, suggestive of paratuberculosis disease, on postmortem examination, when considering the results for both the intestinal homogenate and MLN in parallel. The clinical cases were a subset of the sheep with gross lesions.

The ability of qPCR to detect the presence of MAP in animals with gross lesions was comparable or better than the use of a culture result from both the intestinal and MLN homogenate, which detected 35/38 of the animals. When considering the results separately for the intestinal tissues and MLN of animals with gross lesions, qPCR detected 33/34 intestine culture positive animals and 32/35 MLN culture positive animals. In addition, qPCR of intestinal homogenates detected 2/4 intestinal culture negative animals and qPCR of MLN detected 3/3 MLN culture negative animals.

In addition to confirmation of animals showing clinical signs and gross lesions, qPCR of the tissues detected additional animals as positive for MAP DNA: for the intestinal homogenates, an additional 29 exposed animals were detected, whereas qPCR of MLN homogenates detected an additional 35 animals.

### Ability of the qPCR to Confirm MAP Presence Compared to Tissue Culture and Histopathological Lesion Examination

The qPCR method detected MAP in a majority of tissues identified as infected by the reference tests. For the same sample type, the qPCR tended to detect more samples as MAP positive than culture and histopathological lesion scoring (Table 1). The proportion of test positive results for any of the reference tests compared to the qPCR test for individual tissues was not significantly different when examined by McNemar’s Chi-square test suggesting equivalence of the tests. The agreement when assessed with Cohen’s kappa statistic showed a good to moderate agreement of qPCR with the reference tests.

**Table 1:** Comparison of qPCR test results of tissues (intestine and MLN) with reference test (culture and histopathological lesion score) results.

Sample type	qPCR	Culture			McNemar’s χ^2^ (*p*-value)^c^	Cohen’s kappa (95% CI)^d^	Histopathological lesions				McNemar’s χ^2^ (*p*-value)^c^	Cohen’s kappa (95% CI)^d^
		+^a^	−^b^	Total			+^a^	−^b^	Not done	Total		
Intestine	+	53 (88.4%)	11 (23.5%)	64	0.50 (0.22)	0.66 (0.52, 0.80)	42 (85.8%)	18 (34.0%)	4 (80.0%)	64	4.00 (0.11)	0.52 (0.36, 0.68)
	−	7 (11.7%)	36 (76.6%)	43			7 (14.3%)	35 (66.1%)	1 (20.0%)	43		
	Total	60	47	107			49	53	5	107		
MLN	+	51 (86.5%)	19 (39.6%)	70	3.71 (0.13)	0.48 (0.32, 0.65)	45 (81.9%)	19 (41.4%)	6 (100.0%)	70	2.21 (0.20)	0.42 (0.24, 0.59)
	−	8 (13.6%)	29 (60.5%)	37			10 (18.2%)	27 (58.7%)	0	37		
	Total	59	48	107			55	46	6	107		
Both^e^	+	61 (95.4%)	19 (44.2%)	80	10.23 (0.01)*	NC	52 (89.7%)	23 (52.3%)	5 (100.0%)	80	8.83 (0.02)*	NC
	−	3 (4.7%)	24 (55.9%)	27			6 (10.4%)	21 (47.8%)	0	27		
	Total	64	43	107			58	44	5	107		

*^a^Positive test result. Percentage were calculated as proportion of positive test result of reference test*.

*^b^Negative test result. Percentage were calculated as proportion of negative test result of reference test*.

*^c^McNemar’s χ^2^ at three degree of freedom and corresponding p-value*.

*^d^Cohen’s kappa and 95% CI of the estimate*.

*^e^The parallel test results of both tissues were compared to the parallel test results of reference tests for both tissues*.

*McNemar’s χ^2^ showed ample evidence that the positive proportion of the qPCR of both tissues compared to the reference tests was significantly different.

*NC, not calculated; qPCR, quantitative PCR; CI, confidence interval; MLN, mesenteric lymph node*.

When the tissue qPCR results for both the intestinal and MLN tissues were considered, tissue qPCR detected most of the animals detected by the parallel interpretation of the reference tests (Table 1). In addition, samples that were not detected by the reference tests were detected by the qPCR. Consequently, there was evidence that the qPCR performed better than the reference tests, when assessed with McNemar’s Chi-square test (Table 1).

### Evaluation of Test Performance Parameters of the qPCR Compared to Reference Tests (Culture and Histopathological Lesion Score)

Relative sensitivity of the qPCR assay for intestinal and MLN tissues was determined, assuming tissue culture result or histopathological examination result of the respective tissues as a gold standard (Table 2). The qPCR was more sensitive when assessed against culture than against the histopathological examination result; this was not unexpected as culture and qPCR are both tests for the presence of the MAP organism.

**Table 2:** Performance characteristics of intestinal tissue and mesenteric lymph node (MLN) quantitative PCR (qPCR) with reference to tissue culture and tissue histopathological lesion results.

Test	Tissue	Reference test	Se [95% confidence interval (CI)]^a^	PDLR (95% CI)^b^
qPCR	Intestine	Culture	88.34 (80.22, 96.46)	3.78 (2.24, 6.39)
		Histopathological lesions	85.72 (75.92, 95.52)	2.53 (1.71, 3.74)
	MLN	Culture	86.45 (77.71, 95.18)	2.19 (1.52, 3.15)
		Histopathological lesions	81.82 (71.63, 92.02)	1.99 (1.38, 2.86)
	Both^c^	Culture	95.32 (90.14, 100.00)	2.16 (1.54, 3.04)
		Histopathological lesions	89.66 (81.82, 97.50)	1.72 (1.28, 2.31)

*^a^Se = estimated test sensitivity in percentage and 95% CI of the estimate*.

*^b^Positive diagnostic likelihood ratio and CI*.

*^c^Parallel test results of both tissues were compared with parallel test results of the reference tests for both tissues*.

**Figure 1:**
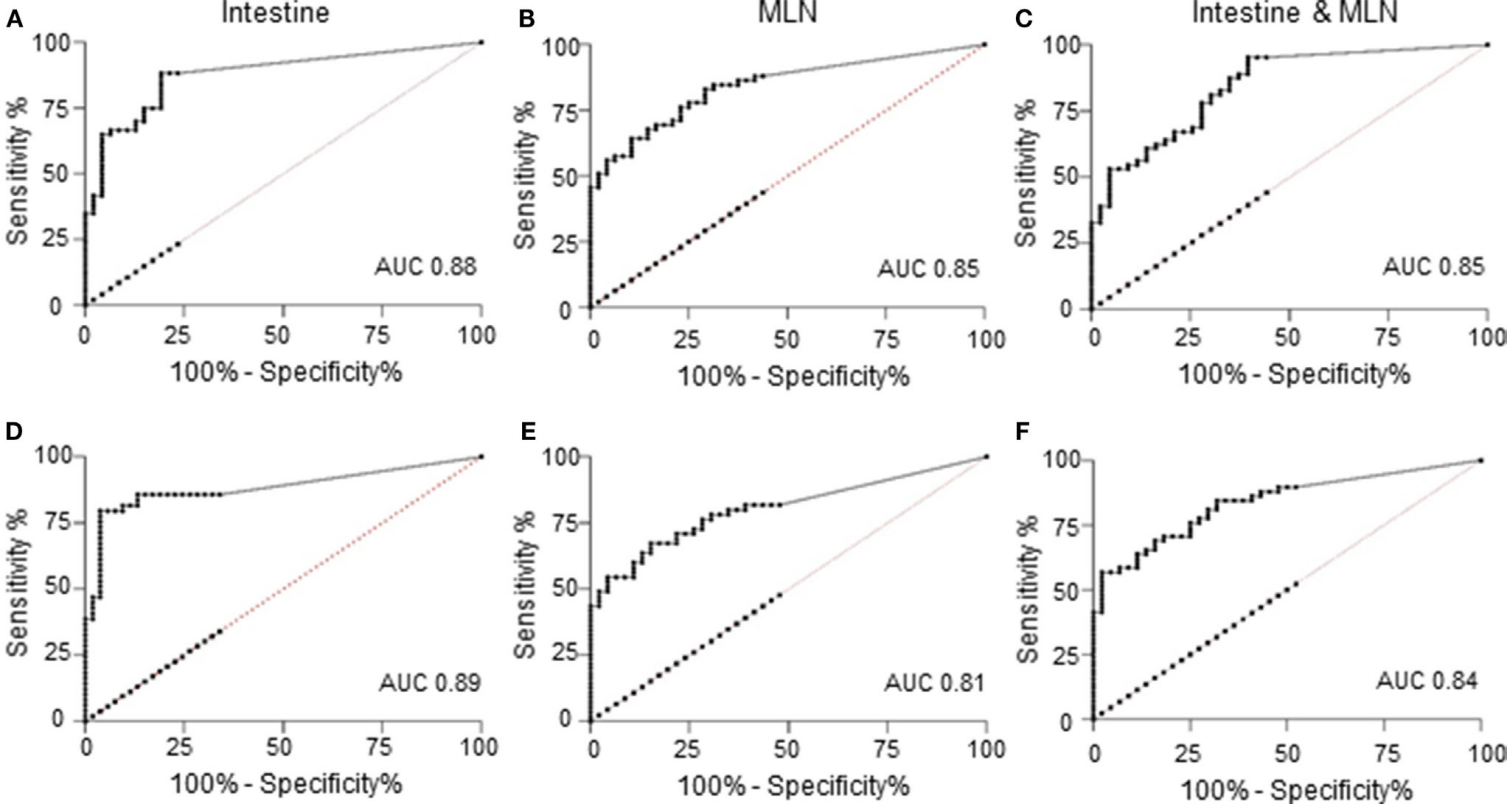
Receiver operating characteristic (ROC) curves of quantitative results of quantitative PCR (qPCR) versus reference tests; tissue culture **(A–C)** or histopathology **(D–F)**. **(A)** ROC curve of qPCR of intestine against culture results as reference test. Area under the curve (AUC) was 0.88 [95% confidence interval (CI): 0.82, 0.95], and at a cutoff of >0.00054 pg, the sensitivity was 88.33% (95% CI: 77.43%, 95.18%), and the likelihood ratio was 4.16. **(B)** ROC curve of qPCR of mesenteric lymph node (MLN) against culture results as reference test. AUC was 0.85 (95% CI: 0.77, 0.92), and at a cutoff of >0.00052 pg, the sensitivity was 86.44% (95% CI: 75.02%, 93.96%), and the likelihood ratio was 2.19. **(C)** ROC curve of qPCR of intestine and MLN against culture results of both tissues as reference test. AUC was 0.85 (95% CI: 0.78, 0.92), and at a cutoff of >0.00053 pg, the sensitivity was 95.31% (95% CI: 86.91%, 99.02%), and the likelihood ratio was 2.28. **(D)** ROC curve of qPCR of intestine against histopathological lesion examination as reference test. AUC was 0.89 (95% CI: 0.82, 0.96), and at a cutoff of >0.00054 pg, the sensitivity was 85.71% (95% CI: 72.76%, 94.06%), and the likelihood ratio was 2.68. **(E)** ROC curve of qPCR of MLN against histopathological lesion examination as reference test. AUC was 0.81 (95% CI: 0.72, 0.89), and at a cutoff of >0.00052 pg, the sensitivity was 81.82% (95% CI: 69.10%, 90.92%), and the likelihood ratio was 1.99. **(F)** ROC curve of qPCR of intestine and MLN against histopathological lesion examination of both tissues as reference test. AUC was 0.84 (95% CI: 0.77, 0.92), and at a cutoff of >0.00053 pg, the sensitivity was 89.66% (95% CI: 78.83%, 96.11%), and the likelihood ratio was 1.80.

A ROC curve analysis showed a good discriminatory power of the qPCR to differentiate between positive and negative results of the reference tests (Figure 1). The positive diagnostic likelihood ratio estimated at the cutoff used for the qPCR (approximately >0.0005 pg) was higher for predicting the culture results compared to the histopathological examination results.

Similarly, when parallel test results of intestinal and MLN qPCR were compared to the parallel test results of culture or histopathological examination, there was a notable increase in sensitivity, with higher sensitivity against culture compared to histopathological lesion score results (Table 2). A ROC curve analysis also showed that parallel use of test results of two tissues was good at discriminating the positive test result of the reference tests when interpreted in parallel (Figures 1C,F).

**Figure 2:**
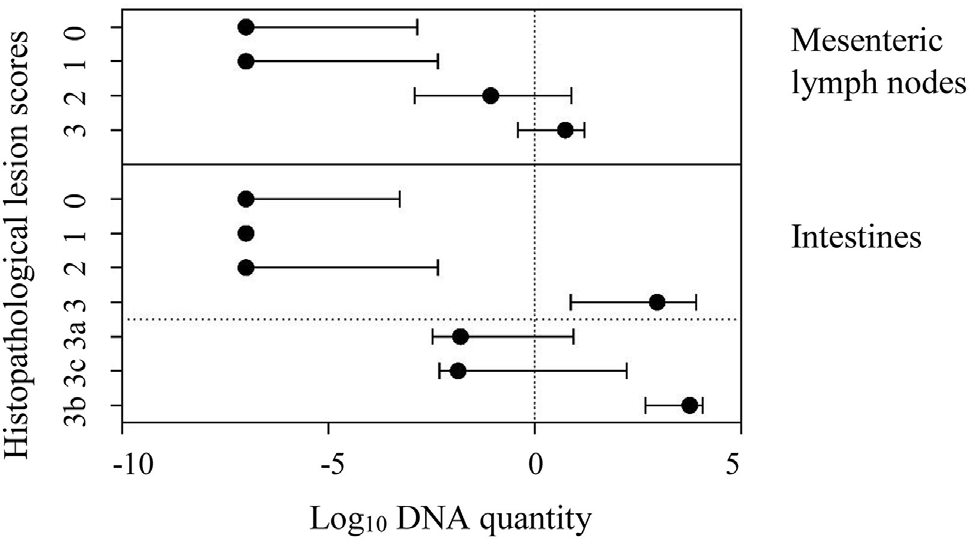
Line plot showing median log_10_ DNA quantity and interquartile range of *Mycobacterium avium* subspecies *paratuberculosis* DNA quantified in mesenteric lymph node and intestinal tissues against the respective histopathological lesion score (according to Pérez et al.) in these tissues. In intestines, the lesion type 3 was further distinguished as subtypes 3a, 3c, and 3b.

The comparison of tissue qPCR with histological lesion score results showed that the number of qPCR positives (Table 3) and MAP DNA quantity (Figure 2) increased according to lesion severity. Furthermore, with each log increase in DNA quantity, the odds of histopathological lesion score of the tissues increased by 1.82 times (Table 4). Likewise, MLN tissues had about half the odds of having a more severe lesion than intestinal tissues, suggesting that lesion scores are more severe in intestinal tissue.

**Table 3:** Comparison of the test result (cutoff ≥0.0005 pg) of intestine and mesenteric lymph node (MLN) quantitative PCR (qPCR) corresponding to various grades of lesion scores on histopathological examination.

Tissues	Histological lesion scores^a^	qPCR result		
		+	−	Total
Intestine	0	18	35	53
	1	0	2	2
	2	2	5	7
	3	40	0	40
	3a	9	0	9
	3b	27	0	27
	3c	4	0	4
	Not done	4	1	5

	Total	64	45	107
MLN	0	19	27	46
	1	5	6	11
	2	26	3	29
	3	14	1	15
	Not done	6	0	6
	Total	70	37	107

*^a^Pérez’s score of grading histopathological lesion*.

**Table 4:** Result of ordinal logistic regression with ordinal histopathological lesion score as response and log_10_ DNA quantified in the quantitative PCR of tissues and types of tissues [intestine and mesenteric lymph node (MLN)] as predictors.

Covariates	Estimate	SE	Odds ratio	*p*-Value
Intercept 1	−1.97	0.35		<0.01
Intercept 2	−0.36	0.32		0.3
Log_10_ DNA (quantitative)	0.60	0.07	1.82 (1.60, 2.07)	<0.01
Tissue type (intestine versus MLN)	−0.85	0.36	0.44 (0.22, 0.87)	0.02

### Comparison of Intestinal qPCR with MLN qPCR

**Figure 3:**
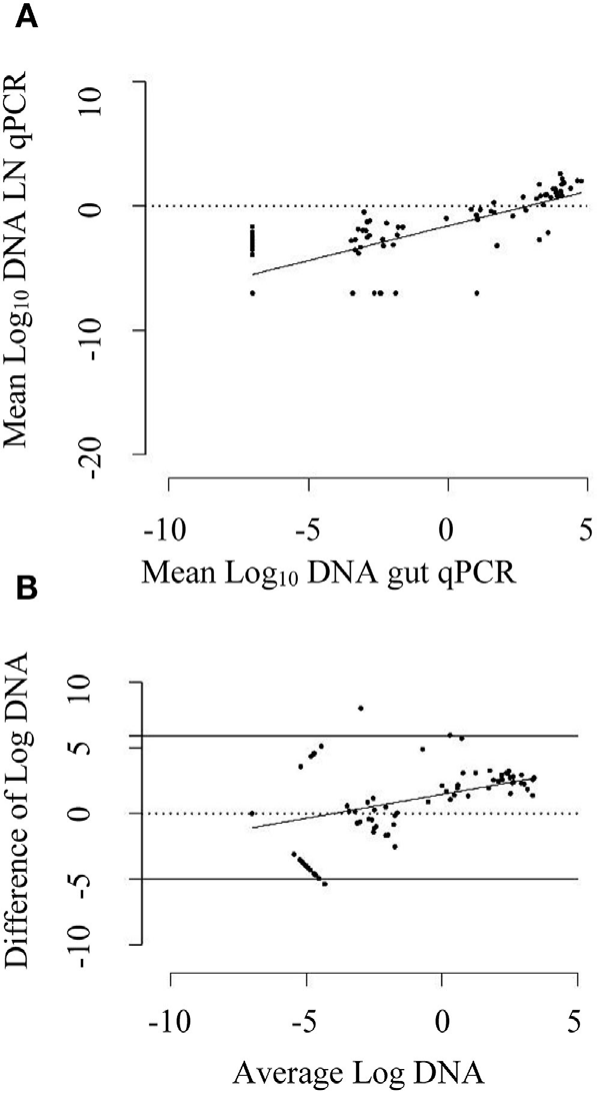
**(A)** Scatter plot of mean log_10_ DNA quantified by mesenteric lymph node (MLN) quantitative PCR (qPCR) against log_10_ DNA quantified by intestinal tissue (gut) qPCR. The solid line is the regression line. A positive correlation coefficient of 0.78 (95% CI: 0.69, 0.84) was observed. **(B)** Bland–Altman plot depicting difference of log_10_ DNA quantified by gut qPCR and MLN qPCR against average log_10_ DNA quantified by them. The dotted line shows the line of perfect agreement whereas two solid lines show the limit of agreement. The DNA quantified by gut qPCR was 2.94 times higher than that quantified by MLN qPCR for the same animal on an average. The solid line is the regression line of average log_10_ DNA against difference of log_10_ DNA. An increase in average difference was observed for the increase in DNA quantity in the gut and MLN homogenate.

A strong positive correlation between DNA quantified by qPCR of intestinal homogenate and MLN homogenates of the same animal was found (Pearson’s correlation coefficient of 0.78, 95% CI: 0.69, 0.84). The DNA quantified by these two tests showed a positive linear relationship (Figure 3A). On an average, MAP DNA levels in the intestine were 2.94 times than those in the associated MLNs. The Bland–Altman graph (Figure 3B) depicts that there was a slight increase in the difference of DNA quantified by intestinal qPCR and MLN qPCR, with an increase in average DNA quantified by these methods.

When the qPCR results from the two tissues (at cutoff point of ≥0.0005 pg) were assessed, the overall agreement between intestine qPCR and MLN qPCR results was good. Although qPCR of MLN homogenates detected more animals compared to qPCR of intestinal homogenates, the proportions of positive test results of the two assays were not significantly different (Table 5). Likewise, the agreement between the two tests was moderate, suggesting the complementary nature of these two tests; animals that went undetected by qPCR test of one sample type were detected by the examination of the other sample type.

**Table 5:** Comparison of quantitative PCR (qPCR) results (cutoff of ≥0.0005 pg/5 µl of DNA extract) of intestinal tissue compared to qPCR results of mesenteric lymph node (MLN) samples.

MLN qPCR result	Intestinal qPCR result			McNemar’s χ^2^ (*p*-value)^a^	Cohen’s kappa [95% confidence interval (CI)]^b^
	Positive	Negative	Total		
Positive	54	16	70	0.97 (0.25)	0.49 (0.32, 0.66)
Negative	10	27	37		
Total	64	43	107		

*^a^McNemar’s χ^2^ at three degree of freedom and corresponding p-value*.

*^b^Cohen’s kappa and 95% CI of the estimate*.

### Strain Typing

**Figure 4:**
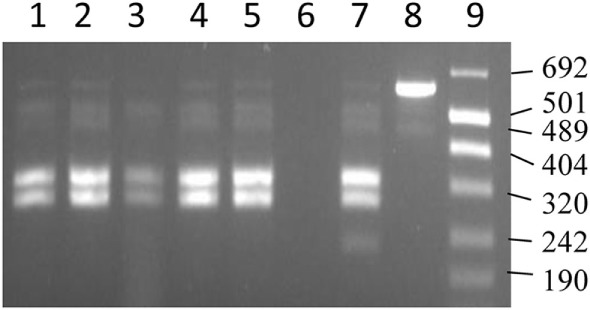
IS*1311* PCR-restriction endonuclease analysis profiles for DNA extracted from tissues. Lanes 1, 2, and 4 are IS*1311* PCR products from DNA extracted from gut tissues restricted with *Mse* I and *Hinf* I enzymes. Lanes 3 and 5 are IS*1311* PCR products from DNA extracted from lymph node tissues restricted with *Mse* I and *Hinf* I enzymes. Lane 6 is negative control. Lane 7 is IS*1311* PCR product from S strain restricted with *Mse* I and *Hinf* I enzymes. Lane 8 is the unrestricted IS*1311* PCR product from S strain. Lane 9 is the molecular size markers (Number VIII).

The good performance of qPCR in identifying infected animals raised the possibility of using the isolated DNA to perform direct strain typing and hence enable rapid molecular epidemiological analysis, essential from a disease control perspective. Strain typing on the directly isolated DNA correctly identified the S-strain of MAP, which was used for the experimental infection of the animals, in the DNA extracted from both intestine and LN tissue of the samples tested (Figure 4).

## Discussion

This study was conducted to investigate whether the relative sensitivity of tissue qPCR compares to tissue culture and histopathological lesion examination to determine the MAP infection status of an animal and predict the potential infectivity of an animal, with a potential application in abattoir surveillance as a replacement test. Furthermore, we investigated whether MAP DNA isolated from intestinal and MLN homogenates could provide rapid information on MAP strain type. The combined information is important in disease investigation and in planning and evaluating OJD management on farms.

It was evident from this study that the qPCR method used gave results that were similar or better than culture results in terms of sensitivity for MAP detection for both of the tissue types assessed. The qPCR test outperformed both culture and histopathological examination tests, when the tests were interpreted in parallel for both tissue types. Moreover, only half of the tissue amount generally used for culture was used for qPCR and the results of qPCR could be obtained within a week compared to 12 weeks required for culture. Both the tests provide similar information on flock health status based on sensitivity and test performance characteristics of the qPCR test. However, the tests could complement each other for the purpose of an individual animal diagnosis, as an animal “missed” by tissue culture could be detected by the tissue qPCR, suggesting that the detection limit and sensitivity of qPCR is better than the culture method, possibly due to the necessary inclusion of a decontamination step in culture methods (30). As has been previously reported, culture tests require the use of a decontamination step that might decrease the diagnostic sensitivity, especially when the contamination level of MAP in the tissue is low (30). Conversely, samples not detected by qPCR, due to low MAP numbers in the tissues, sampling site tested or PCR inhibition, might be detected by culture. PCR inhibition and remediation strategies have been previously reported when assessing fecal sample types (50). Although no obvious evidence of inhibition was found in this study, based on the assessment of the quality of DNA extracts and testing and relief of potential PCR inhibition (50) by performing the qPCR test of fivefold diluted DNA extracts (data not shown), the absence of PCR inhibition cannot be guaranteed as it is a complex phenomenon. Likewise, the inability of qPCR test result to confirm the viability of MAP is a limitation. It was evident in this study that some of the DNA extracts appeared to contain high amounts of initial DNA, possibly target DNA of MAP and other non-target DNA, as determined by the raw baseline fluorescence before amplification. This may be due to the carryover of host DNA, which is expected when bacterial DNA is being extracted from tissues and might result in PCR inhibition (51). However, the qPCR was successful in amplifying the target DNA of MAP, yielding amplification products within the correct melt temperature peak range.

Although the study of histopathological lesions provides different information to that provided by qPCR, the two were on par in their ability to determine MAP infection in the tissues. For this purpose, qPCR detected more animals than histopathological lesion scoring. This is expected as the amount of sample tested in the qPCR is significantly larger compared to that used in preparing the histopathological slides (15). Furthermore, it has also been noted earlier that only in the advanced stage of MAP infection the lesions are diffuse (52, 53), and the sample that goes for DNA extraction and section preparation are mutually exclusive. Likewise, the discrepancy can be contributed to by the disease stage, as was shown when the number of qPCR-positive results were compared by lesion grade; this has been previously noted when comparing tissue culture results with microscopic tissue lesions (15). As lesion severity increased, there was an associated trend toward higher MAP DNA quantities and thus greater likelihood of detection.

The diagnosis of clinical MAP infection is not a problem; most of the available tests can correctly identify animals in the clinical stage of the disease (15). This study also demonstrated that all three types of test (culture, histopathological lesion scoring, and qPCR) were able to diagnose the clinical animal correctly. Although all of the tests were able to detect most of the animals with gross lesions, the qPCR test was similar to or better than the reference tests, including culture and histopathological lesion scoring, for confirming MAP infection, in particular for animals showing the clinical form of the disease or gross lesions on necropsy. However, in addition to the animals showing clinical signs and gross lesions, the qPCR test detected additional non-clinical animals. Presently, slaughterhouse surveillance programs in Australia focus on the examination of histopathological lesions of animals showing gross paratuberculosis lesions in the intestines and MLN (9). This study showed that focusing on gross lesions might miss a lot of animals when assessment of abattoir surveillance was done, as predicted by Abbott and Whittington (8). Similar observations were made in a cattle study (54). This is expected as examination of gross lesions has very low to low diagnostic sensitivity for animal and flock level diagnosis; the flock level sensitivity is particularly low when the flock level prevalence of the disease is very low (8). Furthermore, many animals reaching the slaughterhouse are too young to develop lesions, given the chronic nature of the disease and in particular if the disease prevalence and exposure level is low. It would thus be a good strategy to randomly test intestines and MLNs from slaughtered animals rather than targeting only those animals with gross lesions to obtain a more accurate understanding of MAP infection in a flock or a region. This type of sampling approach would be aided by qPCR techniques that are cost effective and high-throughput and would benefit from a pooling approach subject to validation.

Testing tissue samples from intestines and lymph nodes could complement each other. This study found that, although the agreement between the qPCR results of the two tissue types was very high, it was not perfect. Similar observations of imperfect agreement between the culture results of ileum and MLN have been previously reported (13). This supports the strategy in testing both of these tissues for obtaining broader information on the disease status of the animal. Furthermore, intestinal lesions in naturally and experimentally infected sheep are distributed across the segments of the intestines (52, 53). Likewise, MAP is non-uniformly distributed in the associated MLNs (52). Hence, pooling strategies applied to different sections of intestine and several MLNs, as used in this study, can increase the sensitivity of using intestinal tissue as a sample for diagnosing JD, as observed from our results. As expected, a study in cattle noted an improvement in the detection rate following inclusion of several types of tissues (54). Thus, the pooling strategy and testing of both tissues used in this study detected more animals when compared to testing either of the samples. Furthermore, although the intestinal qPCR result was able to predict the MLN qPCR result, the *R*^2^ estimate was only 60.0% in a regression analysis, suggesting that intestinal qPCR does not completely predict MLN qPCR result. Thus, testing both tissues can give complementary information on the MAP infection status of an animal.

Concerns have been raised over the reduced specificity of qPCR methods for MAP detection, particularly those using IS*900* as the genetic marker of MAP DNA. This was due to published and unpublished reports of IS*900*-like insertion elements in some mycobacterial species (7, 55, 56). The specificity of the PCR primers used in this study had been previously validated against a panel of 51 mycobacterial taxa and strains, including 10 isolates that were reported to contain IS*900*-like elements, with no evidence of false positive test results (7). There have been no other reports of other bacterial species in the gut of sheep that cross react with the IS*900* qPCR. Thus, based on the available information, the results obtained by the current qPCR are very specific and the presence of MAP confirmed by qPCR that went undetected by culture and histopathological lesion scoring is likely due to the increased analytical sensitivity of the current qPCR compared to these tests.

Culture-independent methods using DNA isolated directly from samples have been successfully used for whole genome sequencing, diagnosis, as well as strain typing of mycobacterial species (32, 33). However, the success of such procedures depends on the quality and quantity of the target DNA in the extract and on the typing method. The DNA extracted from the tissues using this method was of high quality (data not shown) and hence was successfully used to strain type MAP by performing the REA method targeting the insertion sequence IS*1311*, previously used to strain type MAP from cultures obtained from tissue sections of sheep (57). The insertion sequence (IS*1311*) is shared by members of *Mycobacterium avium* including MAP and polymorphism in this sequence have been successfully utilized by means of restriction enzymes (*Mse* I and *Hinf* I) to differentiate MAP from *Mycobacterium avium* and to differentiate strains of MAP. Although we did not attempt to make a comparison with different strain typing tools available for MAP (58–60), a study reported that several typing methods complement each other with no significant difference in Simpson’s Index of Diversity when the discriminatory power of different typing methods were assessed (61).

As expected, more DNA was quantified in intestinal samples compared to MLN samples, which indicates the possibility of reliably using MAP DNA isolated from intestinal homogenates to provide rapid information on the MAP strain type and hence perform rapid molecular epidemiological analysis. The popularity of culture-independent methods arises from their efficiency in terms of time, especially when dealing with a fastidious organism like MAP. The culture-independent procedures reported in this study would also help to rapidly diagnose and hence address the concerns of cross-species transmission of MAP strains from sheep to cattle (62). The disease management cost would be higher if culture was required to be used instead for the same information. The classification of MAP as cattle and sheep strain, which is provided by IS*1311* PCR-REA, is sought by epidemiologist to understand the likely source of MAP infection in Australia as the epidemiology of these two strains are considered distinct (63).

The samples from this study came from an infection trial using an experimental infection model, the outcomes of which have been shown to be comparable to what is observed following natural exposure to MAP *via* contaminated pasture (12, 46). Tissue samples are frequently collected and tested in an experimental trial to confirm the infection status of an animal, to understand the disease progression, and to study the dissemination of the MAP infection (27, 52). Tissue samples have also been previously shown to facilitate early detection of MAP infection in sentinel sheep grazed in MAP contaminated pasture with varying level of contamination (12). Tests such as culture and histopathological lesion scoring are used for these purposes. This study shows that validated qPCR methods, where the diagnostic capacity and correlation to other test results are understood, could be equally useful or better for these purposes. Such validated qPCR methods as presented here are equally valuable in disease control and trade certification situations, quantitative risk assessment. Although tissue culture, histopathological lesion scoring, and qPCR tests can complement each other to obtain information on an overall status of the flock, the qPCR can provide rapid information which might be more valuable for rapid interventions and decision making in a disease control and trade dispute scenario. Likewise, when parallel results of qPCR tests of intestine and MLN were used, they provided more information than the parallel results of culture or histopathological lesions. Furthermore, there is the possibility of adopting this test for antemortem diagnosis of high value animals by testing tissue samples obtained *via* biopsy of the animals (17, 20), which is an approach being used in humans (23).

Thus, this study reports a qPCR method for testing intestines and MLNs, validated against the current gold standard method, capable of providing the information currently obtained by testing these sample types using culture and histopathological lesion scoring. As a result, disease status information can be obtained rapidly and used for prompt decision making. Furthermore, the DNA obtained directly from tissues can be used for culture-independent procedures like strain typing. We recommend using both intestine and MLN tissues for testing to improve sensitivity. Further validation of the tissue qPCR method and sampling strategies should be conducted for testing abattoir samples, so that it could replace or complement the currently used surveillance system which is based on gross lesion detection followed by histopathology.

## Ethics Statement

This study protocol was approved by the University of Sydney Animal Ethics committee and the study was carried out in accordance with the recommendations of the Australian code of practice for the care and use of animals for scientific purposes.

## Author Contributions

KA codesigned the study with other authors, developed the protocol, conducted the experiment, analyzed the data, and drafted the manuscript. KP provided laboratory support. ND provided support in analyzing data. RW provided intellectual input during designing and conducting the study. All authors reviewed and revised the manuscript drafts.

## Conflict of Interest Statement

The authors declare that the research was conducted in the absence of any commercial or financial relationships that could be construed as a potential conflict of interest.
